# Supplementary figures and images for: Evolution history of duplicated smad3 genes in teleost: insights from Japanese flounder, Paralichthys olivaceus
Source: PeerJ. 2016 Sep 27;4:e2500. doi: 10.7717/peerj.2500 (PMC5045880; doi:10.7717/peerj.2500)

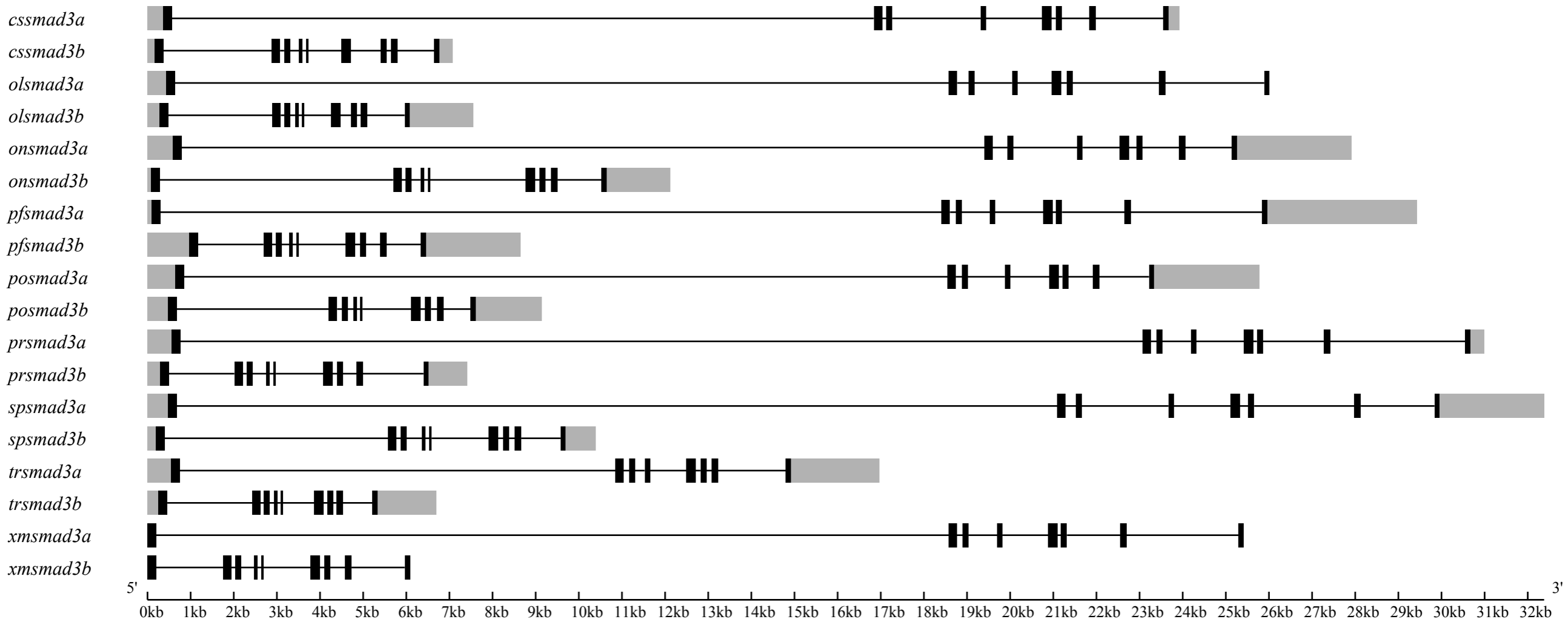

Legend:

■ CDS    ■ UTR    — Intron

Supplement: Supplemental Information 1 — Boxes indicate the exons and the lines indicate the introns. The length of boxes and lines are based on gene length. [file peerj-04-2500-s001.pdf]

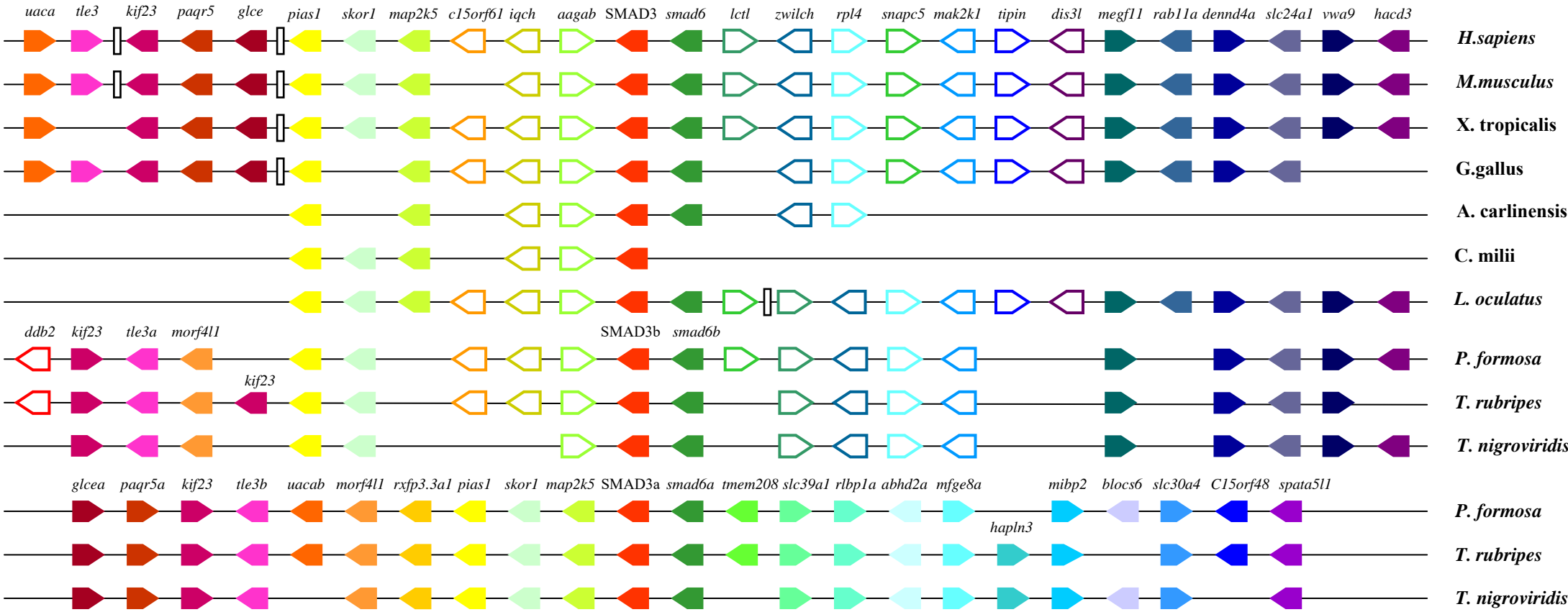

Supplement: Supplemental Information 2 — Different genes are represented by different colored pentagons and gene order is determined according to their relative positions in the chromosome or scaffold; the gene names are placed on top of the pentagons. The direction of pentagons indicates the gene direction, and vertical lines represent noncontiguous regions on the scaffold or chromosome. [file peerj-04-2500-s002.pdf]

**A**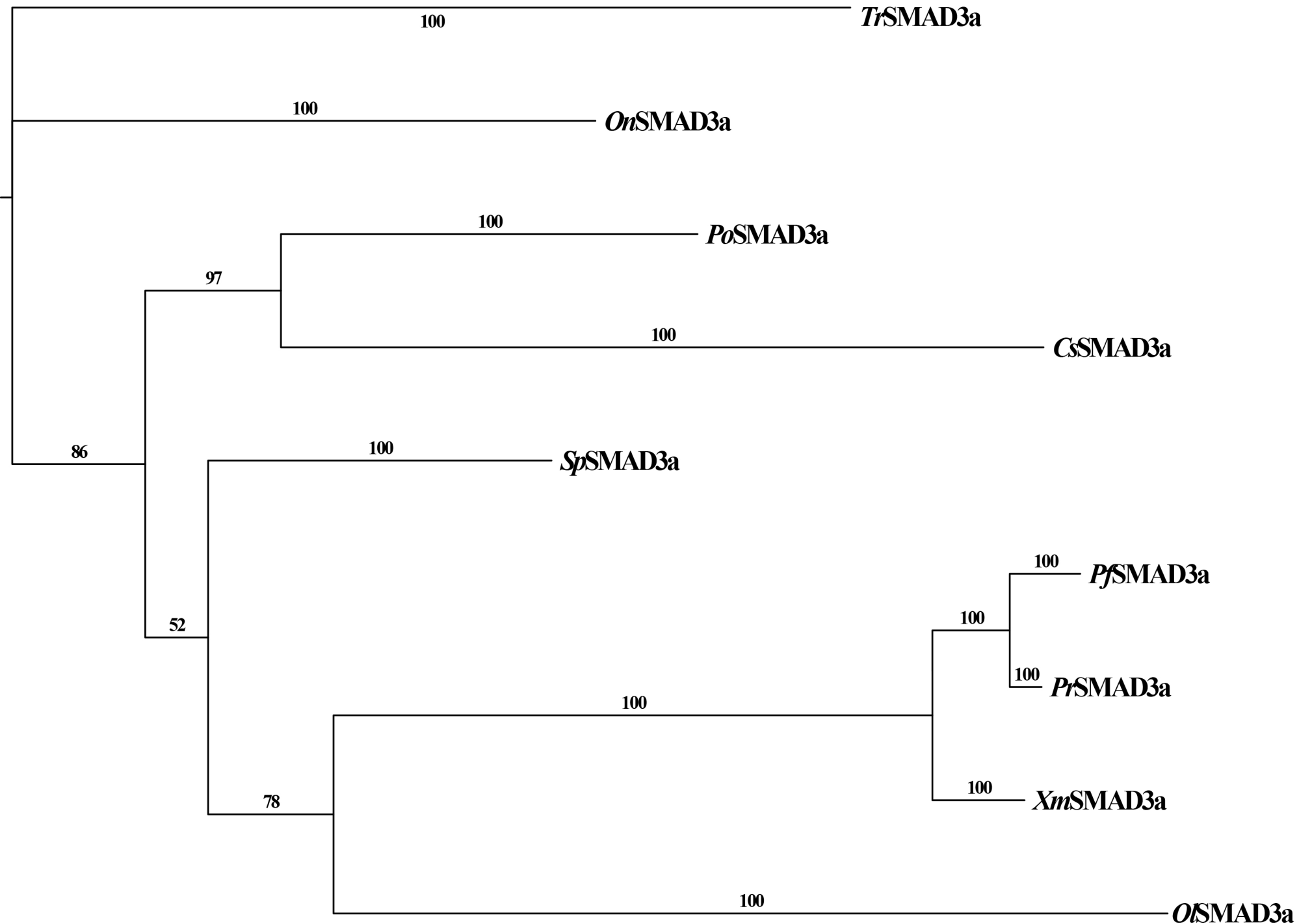

0.02

**B**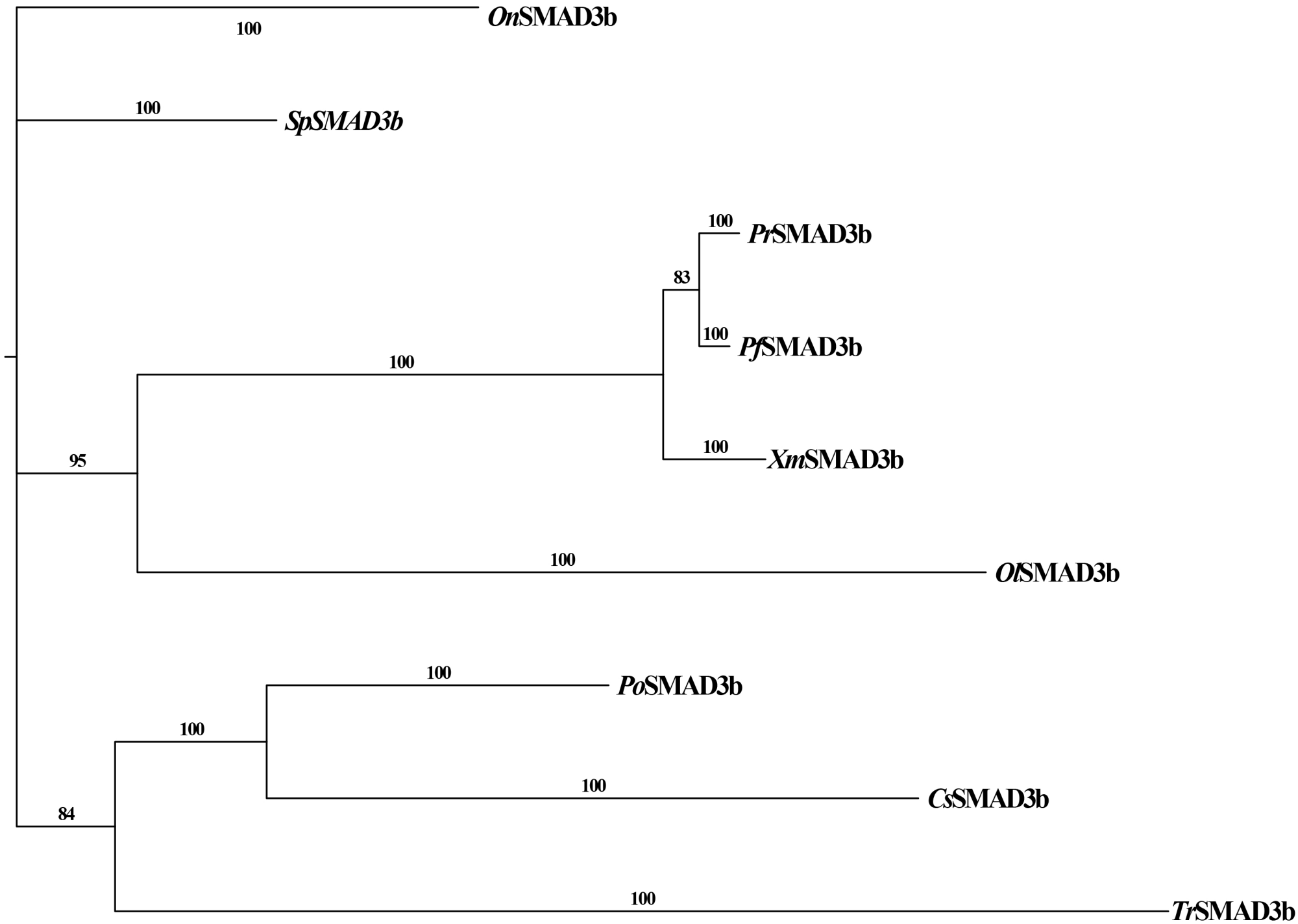

0.02

Supplement: Supplemental Information 3 [file peerj-04-2500-s003.pdf]

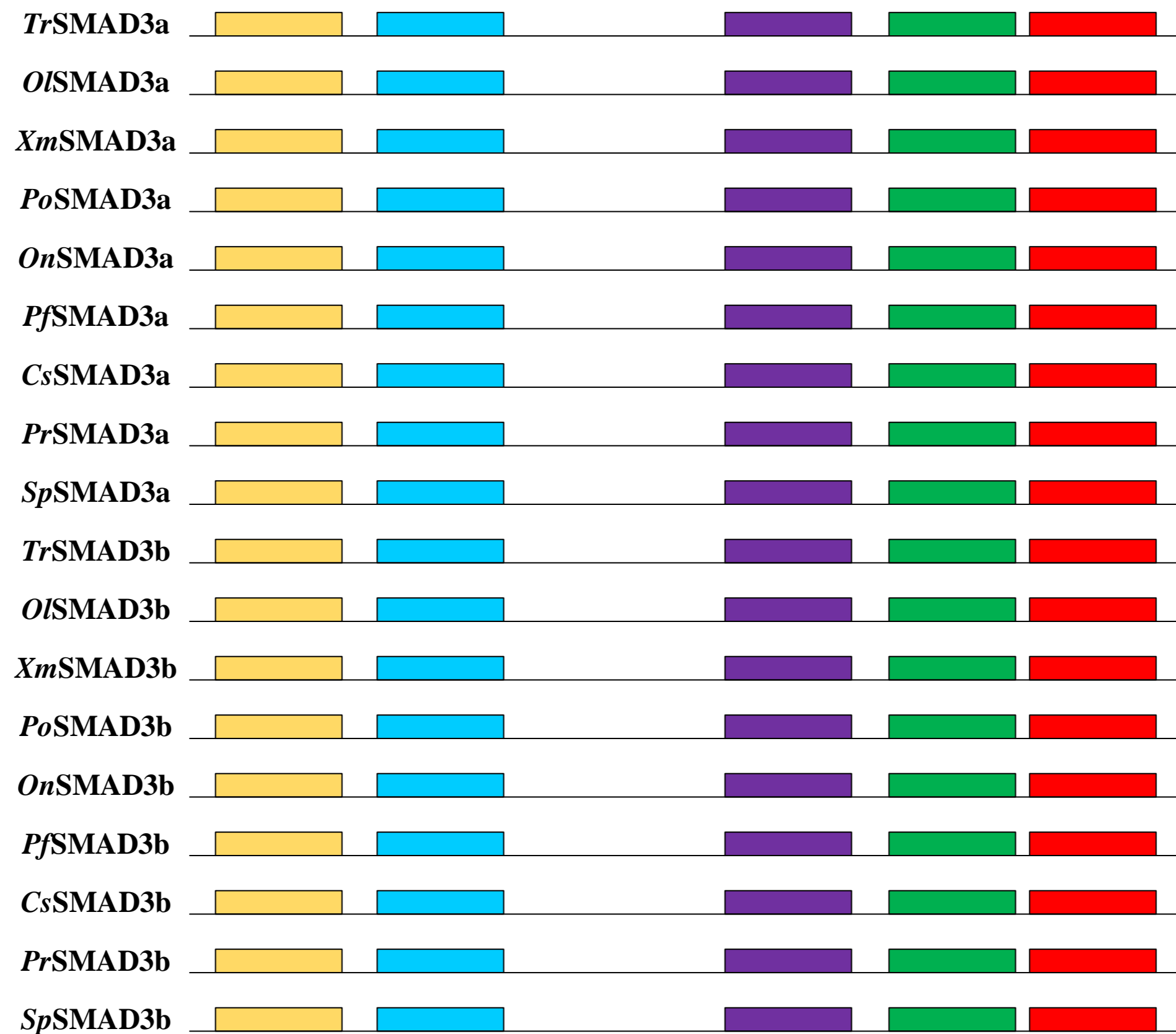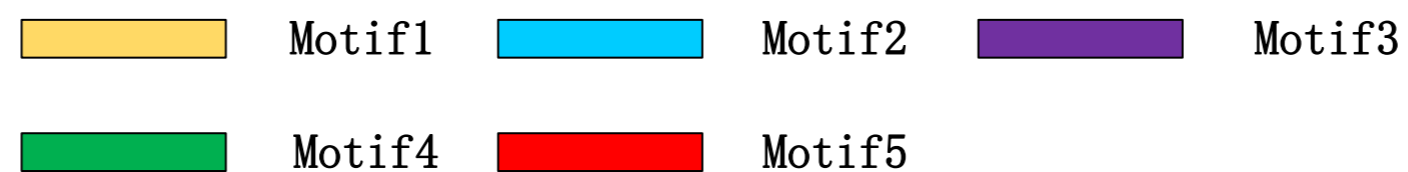

Supplement: Supplemental Information 4 — Tr, Takifugu rubripes; Ol, Oryzias latipes; Xm, Xiphophorus maculatus; Po, Paralichthys olivaceus; On, Oreochromis niloticus; Pf, Poecilia formosa; Cs, Cynoglossus semilaevis; Pr, Poecilia reticulata; Sp, Stegastes partitus. [file peerj-04-2500-s004.pdf]
