# Supplementary material for: Evolution history of duplicated smad3 genes in teleost: insights from Japanese flounder, Paralichthys olivaceus
Source: PeerJ. 2016 Sep 27;4:e2500. doi: 10.7717/peerj.2500 (PMC5045880; doi:10.7717/peerj.2500)
Supplement: Supplemental Information 5 [file peerj-04-2500-s005.docx]

| Common Name | Organism Name | Assembly ID | Gene | Accession No. |
| --- | --- | --- | --- | --- |
| Elephant shark | *Callorhinchus milii* | Callorhinchus_milii-6.1.3 | Smad3 | XM_007910032.1 |
| frog | *Xenopus tropicalis* | Xtropicalis_v7 | Smad3 | NM_001008436.1 |
| Anole lizard | *Anolis carolinensis* | AnoCar2.0 | Smad3 | ENSACAT00000009994 |
| Spotted gar | *Lepisosteus oculatus* | LepOcu1 | Smad1  Smad2  Smad3  Smad4  Smad5  Smad6  Smad7  Smad8 | ENSLOCT00000010824  ENSLOCT00000015308  ENSLOCT00000017172  ENSLOCT00000015878  ENSLOCT00000014175  ENSLOCT00000017160  XM_006627130.2  ENSLOCT00000010329 |
| medaka | *Oryzias latipes* | ASM31367v1 | Smad1 | XM_004065930.2 |
|  |  |  | Smad2a  Smad2b  Smad3a  Smad3b  Smad4a  Smad4b  Smad4c  Smad5  Smad6a  Smad6b  Smad7  Smad8 | XM_004074866.2  XM_004072760.2  XM_00406688.5  XM_004069611.2  XM_004072242.2  XM_004074773.2  XM_004085554.2  XM_004073559.2  XM_011488221.1  XM_004069613.2  XM_004074708.2  XM_004076201.2 |
| Amazon molly | *Poecilia formosa* | PoeFor_5.12 | Smad3a | ENSPFOT00000006410 |
|  |  |  | Smad3b | ENSPFOT00000008837 |
| gubby | *Poecilia reticulata* | Guppy_female_1.0+MT | Smad3a | XM_008404627.1 |
|  |  |  | Smad3b | XM_008412197.1 |
| platyfish | *Xiphophorus maculatus* | Xipmac4.4.2 | Smad3a | ENSXMAT00000002391 |
|  |  |  | Smad3b | ENSXMAT00000003576 |
| bicolor damselfish | *Stegastes partitus* | Stegastes_partitus-1.0.2 | Smad3a | XM_008276051 |
|  |  |  | Smad3b | XM_008276844.1 |
| Nile tilapia | *Oreochromis niloticus* | Orenil1.1 | Smad1 | XM_003449670.3 |
|  |  |  | Smad2a  Smad2b  Smad3a  Smad3b  Smad4a  Smad4b  Smad4c  Smad4d  Smad5  Smad6a  Smad6b  Smad7  Smad8 | XM_003444367.3  XM_003448590.3  XM_003456752.3  XM_003440450  XM_003446059.3  XM_003453862.3  XM_013265612.1  XM_013277587.1  XM_003451806.3  XM_005461566.2  XM_003440449.3  XM_003451503.3  XM_003458254.3 |
| torafugu | *Takifugu rubripes* | FUGU5 | Smad3a | XM_003969638.2 |
|  |  |  | Smad3b | XM_003967452.2 |
| Tongue sole | *Cynoglossus semilaevis* | Cse_v1.0 | Smad3a | XM_008310506.1 |
|  |  |  | Smad3b | XM_008312103.1 |
| human | *Homo sapiens* | GRCh38 | Smad1  Smad2  Smad3 | ENST00000394092  ENST00000262160  ENST00000439724 |
|  |  |  | Smad4  Smad5  Smad6  Smad7  Smad8 | ENST00000398417  ENST00000545279  ENST00000288840  ENST00000262158  ENST00000399275 |
| mouse | *Mus musculus* | GRCm38.p4 | Smad1  Smad2  Smad3  Smad4  Smad5  Smad6  Smad7  Smad8 | ENSMUST00000066091  ENSMUST00000168423  ENSMUST00000034973  ENSMUST00000114939  ENSMUST00000069557  ENSMUST00000041029  ENSMUST00000026999  ENSMUST00000029371 |
| Chicken | *Gallus gallus* | Galgal5 | Smad1  Smad2  Smad3  Smad4  Smad5  Smad6  Smad7  Smad8 | XM_015276130.1  NM_204561.1  NM_204475.1  XM_015298563.1  XM_015293966.1  NM_204248.1  XM_427238.5  XM_015277641.1 |
| Japanese flounder | *Paralichthys olivaceus* | unpublished | Smad1 |  |
|  |  |  | Smad2a  Smad2b  Smad3a  Smad3b  Smad4a  Smad4b  Smad4c  Smad4d  Smad5  Smad6a  Smad6b  Smad7  Smad8 |  |
