# Supplementary material for: Evolution history of duplicated smad3 genes in teleost: insights from Japanese flounder, Paralichthys olivaceus
Source: PeerJ. 2016 Sep 27;4:e2500. doi: 10.7717/peerj.2500 (PMC5045880; doi:10.7717/peerj.2500)
Supplement: Supplemental Information 6 [file peerj-04-2500-s006.docx]

| Primers | Sequence(5’-3’) | Usage |
| --- | --- | --- |
| poSmad3a-FW | TGCGAGGTAAAGACCTTGTG | qPCR |
| poSmad3a-RV | AGTAAACCAACCACGGACTG | qPCR |
| poSmad3b-FW | GACGCCATTCGTCAAGTATCT | qPCR |
| poSmad3b-RV | TACGGCAAATACCCTGGTTG | qPCR |
| 18S-FW | GGTAACGGGGAATCAGGGT | qPCR |
| 18S-RV | TGCCTTCCTTGGATGTGGT | qPCR |
